# Paroxysmal supraventricular tachycardia as a major clinical presentation of the primary coronary sinus lymphoma: A case report

**DOI:** 10.1097/MD.0000000000024225

**Published:** 2021-01-08

**Authors:** Ling Wang, Lixia Cai, Xiangyu Chen, Zhelan Zheng

**Affiliations:** a Department of Ultrasound, Zhuji People's Hospital of Zhejiang Province, Zhuji; b Department of Ultrasound, The First Affiliated Hospital of Zhejiang University, Zhejiang; c Department of Ultrasound, Zhuji People's Hospital of Zhejiang Province, Zhuji Affiliated Hospital of Shaoxing University, Zhuji, China.

**Keywords:** cardiac lymphoma, coronary sinus, echocardiography, supraventricular tachycardia

## Abstract

**Rationale:**

Primary cardiac lymphoma is a rare tumor, especially a tumor located in coronary sinus (CS). The most common symptom of cardiac tumors is dyspnea, accounting for 64%, followed by chest pain, accounting for 26%. However, the cases with paroxysmal supraventricular tachycardia (SVT) as a major clinical presentation are extremely rare.

**Patient concerns:**

We report a 55-year-old female patient with primary CS lymphoma and paroxysmal SVT.

**Diagnoses:**

After the surgical resection, pathology revealed the evidence of diffuse large B-cell lymphoma.

**Interventions:**

The patient underwent chemotherapy after CS tumor resection.

**Outcomes:**

The patient was disease-free during the 6-month follow-up.

**Lessons:**

CS enlargement may be the cause of SVT. Echocardiography should focus on the CS section to arrive at the right diagnosis.

## 1 Introduction

Primary cardiac tumors are uncommon, with an autopsy diagnosis rate of about 0.02%; about 25% of cardiac tumors are malignant,^[1]^ with 95% of malignant tumors being sarcomas, and the other 5% are lymphomas.^[2]^ Primary cardiac lymphomas (PCLs) are extremely rare. In addition, 92% of cardiac lymphomas involve the right atrium or right ventricle, whereas only 7% involve the left ventricular system,^[3]^ and only 1 case has been reported involving coronary sinus (CS).^[4]^ The most common symptom of cardiac tumors is dyspnea, accounting for 64%, followed by chest pain, accounting for 26%.^[5]^ However, the cases with supraventricular tachycardia (SVT) as a major clinical presentation are extremely rare. As the survival period of PCL is only 7 months, and most patients die within a couple of months after diagnosis, early diagnosis and treatment of PCL are extremely important.^[6]^ Hereby, we report a case of primary CS lymphoma and review the literature, with focus on the mechanism of its arrhythmia and the significance of imaging investigations for disease diagnosis in clinical practice.

## 2 Case report

**Figure 1:**
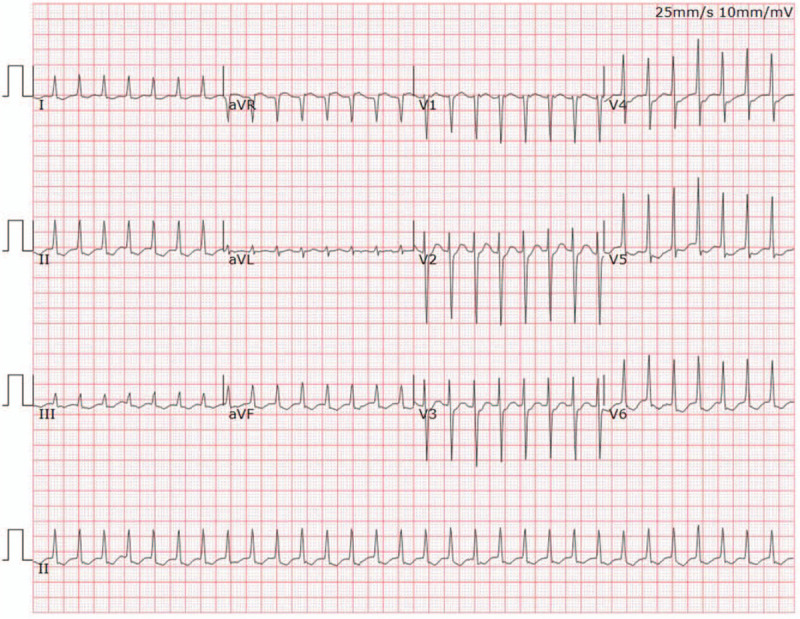
Electrocardiogram showed paroxysmal supraventricular tachycardia.

**Figure 2:**
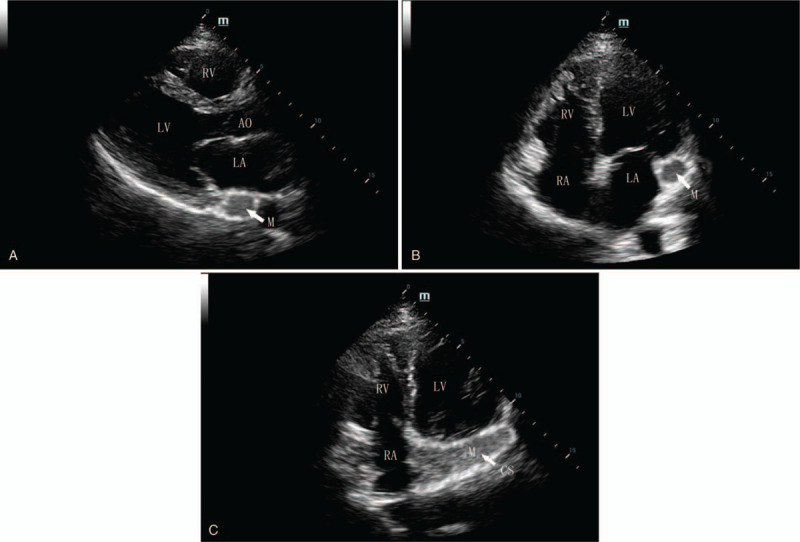
(A) TTE showing a dilated coronary sinus (CS) filled with a hypoechoic mass in the parasternal long axis view. (B) The CS tumor was detected in the apical four-chamber view. (C) A low echo mass extended from the CS to the right atrium in the non-standard four-chamber view. AO = aorta, CS = coronary sinus, LA = left atrium, LV = left ventricle, M = mass, RA = right atrium, RV = right ventricle, TTE = transthoracic echocardiography.

**Figure 3:**
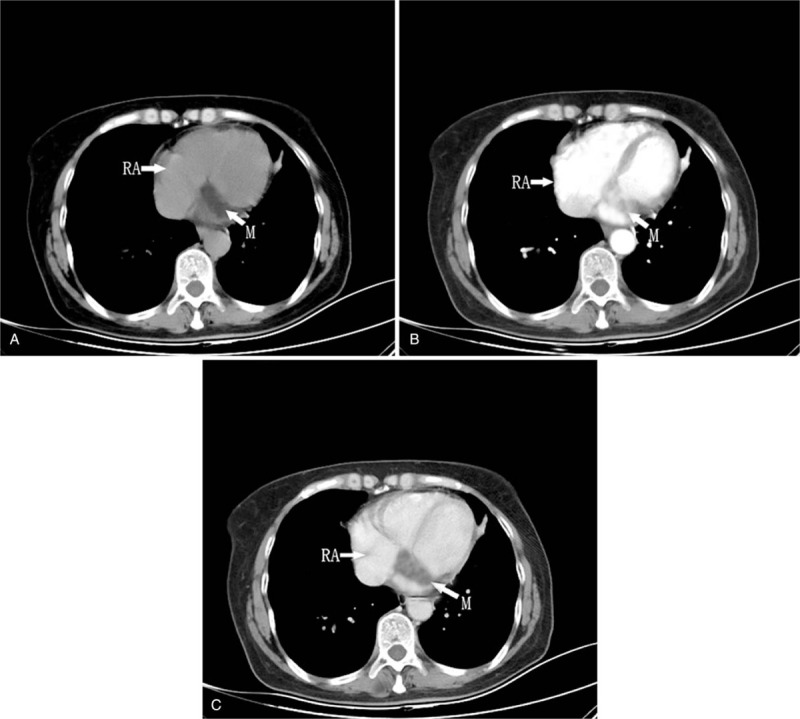
(A) Computed tomography of a low-attenuating lesion in the coronary sinus before contrast agent injection. (B) The tumor displayed heterogeneous moderate enhancement during the arterial phase. (C) Heterogeneous mild enhancement of the tumor in the delayed phase. M = mass, RA = right atrium.

**Figure 4:**
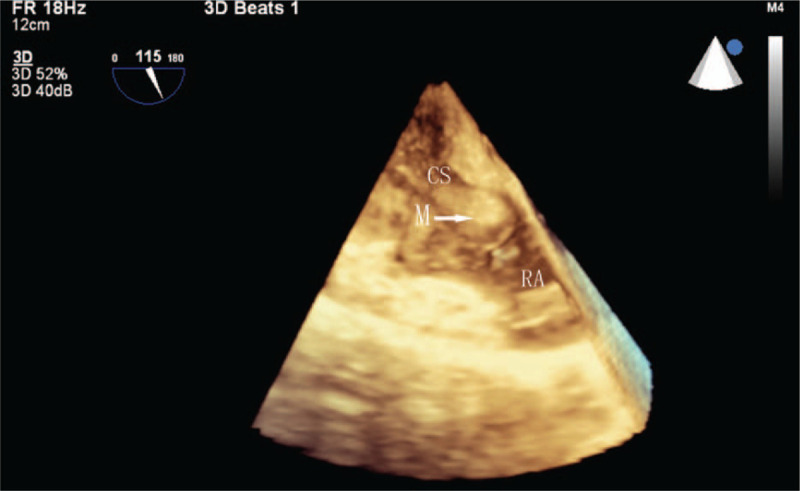
Three-dimensional transesophageal echocardiography showed the morphology of the mass. CS = coronary sinus, M = mass, RA = right atrium.

**Figure 5:**
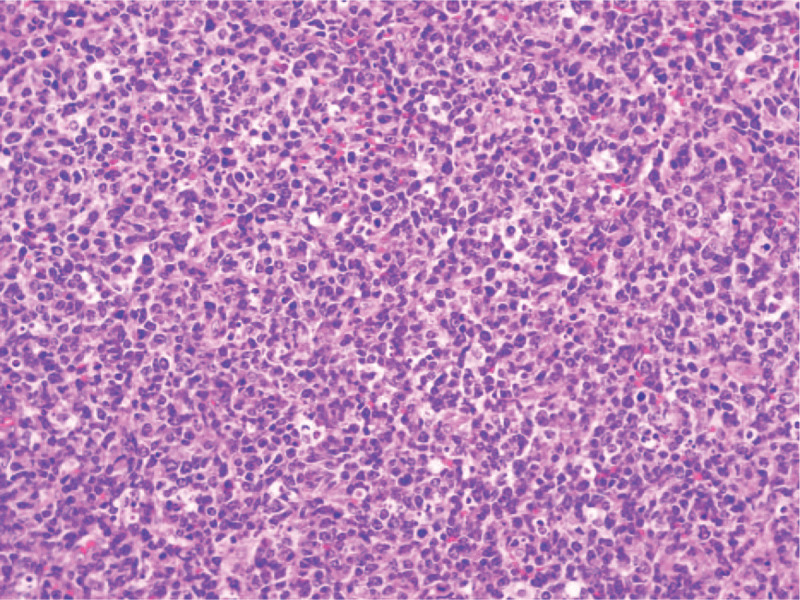
Photomicrograph of the tumoral tissue showed diffuse large B-cell lymphoma.

A 55-year-old woman presented with palpitations of a month's duration, and occasionally had shortness of breath after exertion. Arrhythmia was diagnosed by the local community hospital. She was referred to our hospital for a further comprehensive examination. Her blood pressure was 119/70 mmHg. Her body temperature was 36.9°C and respiratory rate was 19 times per minute. The oxygen saturation was 98%, auscultation showed no murmur and her heart rate was about 120 bpm with regular rhythm. The electrocardiogram (ECG) showed paroxysmal SVT (Fig. 1). A series of imaging investigations were performed after reducing rapid heart rate by medication. Transthoracic echocardiography (TTE) showed that a dilated CS was filled with a lobulated hypoechoic mass in the parasternal long axis view (Fig. 2A). No enlargement of each cardiac cavity was found in the 4 chambers of the heart view (Fig. 2B). In the nonstandard 4-chamber view of the CS, there was a heterogeneous hypoechoic mass that extended from the CS to the right atrium (Fig. 2C). The left ventricular relaxation was decreased, and the ejection fraction was in the normal range. Before contrast agent injection, contrast-enhanced computed tomography (CECT) revealed a low attenuation in the CS (Fig. 3A). During the arterial phase, the tumor showed heterogeneous moderate enhancement (Fig. 3B). Heterogeneous mild enhancement of tumor was displayed in the delayed phase (Fig. 3C). The white blood cell count was 6.4 × 10^9^ cells/L, neutrophils (70.4%), and C-reactive protein level (1.7 mg/L). The possibility of infection was excluded. The d-dimer was 128 μg/L, eliminating the possibility of thrombosis. HIV test was negative. Hepatitis B and C and tuberculous testing were negative. No metastasis was detected in other organs by positron emission tomography. Therefore, there was a high likelihood that this mass was a primary malignant tumor, recognized based on TTE and CT imaging features. To prevent cardiac complications, we performed an emergency surgery to remove the tumor. We utilized echocardiography to monitor the surgical process. The mass was ellipsoidal in 3-dimensional transesophageal echocardiography. The boundary of the mass was clear. The vessel wall of CS was not damaged and the surrounding structure was not involved **(**Fig. 4). The volume of the mass was 13.5 cm^3^. The resected tissue was a pile of gray-brown broken tissue. Pathological examination showed diffuse large B-cell lymphoma with expression of CD20, Ki-67 (+,80%), CD30, Bcl-2, Bcl-6, PAX-5, CyclinD1, c-Myc (+,30%), CD23 (FDC+) (Fig. 5**)**. No complications occurred after the operation, and no abnormality was found on the repeat ECG. Bone marrow biopsy demonstrated no evidence of lymphoma involvement. The disease was considered to be an extranodal marginal zone lymphoma of Ann Arbor stage I_E_. The patient subsequently received chemotherapy. Six cycles of chemotherapy with rituximab, cyclophosphamide, epirubicin, vincristine, and prednisone (R-CHOP) were completed. Thereafter, the patient was followed up for 6 months after the last cycle of R-CHOP and found to be completely asymptomatic.

## 3 Discussion

PCL is extremely rare, accounting for only 1.3% of primary cardiac tumors and only 0.5% of lymphomas.^[7]^ To the best of our knowledge, only 2 primary CS lymphomas have been reported, including this presented case. Our case is the only one with paroxysmal SVT as a major clinical presentation. Another case was characterized by dyspnea and restricted left ventricular filling disorder.^[4]^ Our patient only showed abnormal left ventricular relaxation, which may be related to diversity of the CS pressure caused by the difference in size of the 2 tumors.^[8]^ Neither of the cases presented heart failure or pericardial effusion.

Okumura et al^[9]^ assessed the anatomy of the CS through intracardiac echocardiography and reported that the patients with SVT had a wider CS diameter and area. Senturk et al^[10]^ used 3-dimensional echocardiography to measure the CS and found that the diameter and area of the CS in SVT patients were larger than those without SVT. Weiss et al reported that with CS diameter increased, a diverticulum, or a tumor-like dilatation may stretch surrounding the normal atrial tissue and change the conduction characteristics of the periosteal tissue. Therefore, it may create a decelerated potential area which increased stretching might change the electrophysiological characteristics of the cardiac tissue. CS enlargement, which is caused by CS tumors, may be the reason for paroxysmal SVT.

It was noticed that the patients presenting with rhythm disturbances, survived for a longer time than those with normal rhythms.^[5]^ Kim et al^[11]^ reported a case where the initial diagnosis only showed one degree of atrioventricular block 7 years before and no intracardiac mass was found on echocardiography, with occurrence of various cardiac arrhythmias during follow-up. Seven years later, a giant right lymphoma was detected. The detection of arrhythmia upon presentation may have led to earlier treatment of the affected population. This could explain the reason why PCL patients with arrhythmias have a longer survival time. The case reported hereby was disease-free at the 6-month follow-up, which may be related to the early detection of lymphoma. However, due to the poor prognosis of cardiac lymphoma, this patient needs longer time of follow-up.

CECT can show the blood supply characteristics of the tumor. Bai et al^[12]^ reported that CECT showed mild-to-moderate enhancement of diffuse large B-cell lymphoma, which was similar to our case. Compared with CT, echocardiography does not have the risk of radiation and contrast agent allergy. TEE is useful for evaluation of the location, size, shape, and mobility of a cardiac tumor, with its sensitivity up to 97%.^[13]^ Although magnetic resonance imaging (MRI) can be used to visualize the anatomy of the CS and measure its blood flow, the quality of MRI may be poor due to the patient's rapid heart rate.^[14]^ Therefore, the preferred method of examination for this patient is echocardiography. Physicians need to be aware that CS is not included in conventional echocardiographic views, and there is the possibility of CS anatomical variation which can easily lead to missing the diagnosis.^[15]^ Therefore, for suspected patients, careful observation of CS is required.

Until now, chemotherapy, radiotherapy, and surgery are the main treatments for PCL, and systemic chemotherapy is still the most fundamental therapy for the disease. In our report, the resection of CS tumor relieved patient's arrhythmia and the systemic chemotherapy prevented disease from recurring in the short follow-up time. However, the prognosis of PCL is very poor with a 10% survival rate in 9 to 12 months without any treatment, and earlier diagnosis may improve prognosis.^[16]^

## 4 Conclusions

PCL lacks the specific clinical symptom, and various arrhythmias may be an early presentation. Surgical removal of tumor is usually indicated to avoid catastrophic complications. The utilization of echocardiography as a diagnostic modality can help in establishing the diagnosis and guide the surgical resection.

## Acknowledgments

The authors are grateful to all physicians contributing data to this study.

## Author contributions

**Conceptualization:** Xiangyu Chen.

**Data curation:** Xiangyu Chen, Zhelan Zheng.

**Software:** Zhelan Zheng.

**Writing – original draft:** Ling Wang, Xiangyu Chen.

**Writing – review & editing:** Xiangyu Chen, Lixia Cai.
